# A Useful Strategy to Evaluate the Quality Consistency of Traditional Chinese Medicines Based on Liquid Chromatography and Chemometrics

**DOI:** 10.1155/2015/589654

**Published:** 2015-11-04

**Authors:** Pei Wang, Lei Nie, Hengchang Zang

**Affiliations:** ^1^School of Pharmaceutical Sciences, Shandong University, Jinan 250012, China; ^2^Center for Excellence in Post-Harvest Technologies, North Carolina Agricultural and Technical State University, North Carolina Research Campus, 500 Laureate Way, Kannapolis, NC 28081, USA

## Abstract

Evaluation of the batch consistency of traditional Chinese medicines (TCMs) is essential for the promotion of the development and quality control of TCMs. The aim of the present work was to develop a useful strategy via liquid chromatography and chemometrics to evaluate the batch consistency of TCM preparations. Xin-Ke-Shu (XKS) tablet was chosen as a model for this method development. Four types of chromatographic fingerprint approaches were compared by using similarity analysis based on cosine of angel or correlation coefficient. Differences in the fingerprints of 71 batches of XKS tablet were illustrated by hierarchical cluster analysis. Then, Mahalanobis distance was employed for estimating the probability level (*P* < 0.05) of the differences mentioned above. Additionally, *t*-test was applied to find out the chromatographic peaks which had significant differences. For XKS tablet, the maximum wavelength fingerprint had the largest range and dispersion degree of similarity as compared with the other three ones. There were two clear clusters in all the batches of samples. And we clearly arrived at the conclusion that higher similarity does not exactly indicate small Mahalanobis distance, while lower similarity indicated larger Mahalanobis distance. Finally, a useful strategy was proposed for evaluation of the batch consistency of XKS tablet.

## 1. Introduction

Traditional Chinese medicines (TCMs), also known as botanical medicines or phytomedicines, have been used to promote health and treat diseases for over a millennium. Differing from synthetic drugs, medicinal herbs and their preparations exert therapeutic effects based on the synergic effects of multicomponents involved. Because of low toxicity and effective therapeutic performance, a rapid growth in worldwide demand for TCMs has appeared in the last few decades, especially in Asia, Europe, and North America. As demand grows, the requirement for product quality is also improving. Each batch of TCMs should meet certain product specifications including both the time of production and over its shelf life. However, the quality control for TCMs is more difficult than that for synthetic drugs, because even a simple preparation consisting of only a few herbs may comprise hundreds of mostly unique or species-specific compounds. Complete characterization of all the compounds in a preparation is indeed a challenge [1], while selection of several compounds for determining either efficacy or quality is not in line with the principle of TCMs [2]. In recent years, significant efforts have been made to develop new analytic methods for quality control of TCMs. Among all quality control methods, chromatographic fingerprint is a generally accepted technique for the quality assessment of TCMs.

Chromatographic fingerprint, as a unique pattern indicating the information on multiple chemical components within a sample, could provide more informative and accurate assessment of complex matrix, such as TCM. In order to promote the valid fingerprint method for the quality control of herbal medicines, the China Food and Drug Administration (CFDA) suggested that all of herbal chromatograms should be evaluated by similarity [3], which is based on the calculation of correlative coefficient and/or cosine of vectorial angle (i.e., congruence coefficient) between fingerprint data [4]. Now similarity evaluation is widely used as a close measure for assessment of chromatographic fingerprints in practice. However, similarity based on correlative coefficient or cosine of vectorial angle is the overall evaluation of fingerprints; it is ambiguous and insensitive to the changes in content of components [5]. That is, the similarity between chromatographic fingerprints could not sufficiently indicate the closeness in the contents of components [6], especially for low-content components. Recently, HPLC fingerprinting combined with quantitative determination of some characteristic compounds has been developed and validated for quality control of herbal preparations [7]. A similar conclusion was drawn in the combinative method. Although sample fingerprints shared a satisfactory similarity, the contents of some compounds between samples may still appear quite different (i.e., similar fingerprints do not mean similar components in content) [8]. This may indicate that the slight difference in similarity between fingerprints could suggest the large difference in amounts of components. It is unclear whether the difference in fingerprint similarity is significant or not. How to assess the difference in fingerprint similarity is worthy of investigating. To ensure the quality of TCMs, quantitative determination of as many constituents as possible is quite necessary. But for now it is unrealistic to identify and quantify all the chemical components in such a complex system as TCM. Therefore, a rational strategy for evaluating the consistency of fingerprints is very necessary.

The Xin-Ke-Shu (XKS) tablet is one of the most commonly used tablets in TCMs for treatment of coronary heart and cerebrovascular disease. It is a really complex matrix, which comprises five medicinal materials or extracts thereof, including* Salviae Miltiorrhizae* Radix et Rhizoma,* Pueraria lobatae* Radix,* Crataegi* Fructus,* Notoginseng* Radix et Rhizoma, and* Aucklandiae* Radix. Due to a high complexity in chemical composition, the quality evaluation of XKS tablet is quite a difficult work. So far quite a few approaches have been developed [9]. In our recent work, we developed a high performance liquid chromatography-diode array detector (HPLC-DAD) method for quantification of multiconstituent in XKS tablet and found that the component contents in different batches of XKS tablet had large differences [6].

With the development of chemometric methods, a number of data processing tools were introduced and applied to process chromatographic fingerprints [10–13]. In our present study, chromatographic fingerprint combined with chemometric methods was developed for batch consistency evaluation of XKS tablet. Firstly, four types of fingerprints characterized by different modes were used and compared by similarity analysis. According to the results of comparison, the representative fingerprint was selected for further investigation. Then, hierarchical cluster analysis was applied to observe whether class difference occurred in different batch samples. If high fingerprint similarity was achieved, but apparent class difference existed in samples, then Mahalanobis distance analysis was employed to estimate the probability level of obviously different samples. The relationship between similarity analysis and Mahalanobis distance was also discussed. Subsequently, for the significantly different fingerprints of batches determined by Mahalanobis distance, *t*-test was used to find the peaks with significant difference in areas. Although these peaks found by *t*-test were based on peak areas, the results were theoretically identical to those based on concentration. The corresponding interpretation in theory was also provided. The peaks having significant differences were identified by HPLC/IT-MS^*n*^ as well. Finally, a useful strategy based on the combination of HPLC fingerprint and chemometric methods was proposed for rational evaluation of batch-to-batch consistency of XKS tablet and could be also useful for assessing the batch consistency of other TCMs.

## 2. Methods

### 2.1. Materials and Reagents

HPLC-grade acetonitrile and methanol (MeOH) were purchased from Merck Company (Merck, Darmstadt, Germany). Ultrapure water was purified with a Milli-Q system (Millipore, Bedford, USA). Formic acid (HPLC grade) was purchased from Tedia Company Inc. (Tedia Way, Fairfield, USA). Reagent grade MeOH was purchased from Tianjin Kemiou Chemical Limited Company (Tianjin, China). In order to include as many sources of sample variability as possible, seventy-one batches of XKS tablet that were produced in a long time span of three years were provided by Shandong Wohua Pharmaceutical Limited Company in China. All the XKS tablet samples were kept in a cool room at 4°C and 30% relative humidity with packages; storage stability of XKS tablet, both the physical and chemical properties, was validated. In the cool room, the chemical profiles and the concentration of the major compounds (we determined 6 of the major compounds in XKS tablet, details not shown here) of XKS tablet were consistent for 3 years.

### 2.2. Preparation of Sample Solutions

The outer coats of XKS tablets were removed. All the samples were milled to the homogeneous size. The pulverized powder of 100 mg for each sample was accurately weighed and ultrasonically extracted with 5 mL 70% methanol for 20 min in a conical flask at room temperature. This sample solution (4 mL) was transferred into 10 mL centrifuge tube and centrifuged at 3000 rpm for 10 min. The supernatant was further filtered through a 0.45 *μ*m membrane filter before HPLC injection.

### 2.3. Instrumentation and Chromatographic Procedures

An Agilent 1200 series HPLC system (Agilent Corp., Santa Clara, CA, USA) with a diode array detector (DAD) was used to acquire chromatograms and UV spectra. All of the chromatographic analysis was performed on a Phenomenex C_18_ column (250 mm × 4.6 mm i.d. 5 *μ*m) protected by a precolumn (12.5 mm × 4.6 mm i.d. 5 *μ*m) of the same material. The mobile phase was composed of acetonitrile (A) and 0.1% formic acid in water (B). The conditions of solvent gradient elution were 8–12% (A) in 0–21 min, 12–17% (A) in 21–31 min, 17–38% (A) in 31–55 min, 38–90% (A) in 55–65 min, and 90% (A) in 65–75 min, at a flow rate of 1.0 mL·min^−1^. A preequilibration period of 10 min was set between individual runs. The UV detector was set at 278 nm with full spectral scanning from 190 nm to 400 nm at 2 nm step. The column temperature was maintained at 30°C, and all the injection volumes of sample solutions were fixed at 10 *μ*L.

An Agilent 6310 ion trap mass spectrometer (Agilent Corp., Santa Clara, CA, USA) was connected to HPLC system via an ESI interface. Ultrapure helium (He) was used as the collision gas, and pure nitrogen (N_2_) was employed as the nebulizing gas. The instrumentation and chromatographic condition of HPLC for LC-MS^*n*^ were the same as those used for HPLC-DAD analysis. For identification of the interesting constituents in the fingerprints of XKS tablet, the following conditions of MS analysis were used: negative ion mode; capillary voltage 3.5 kV; drying gas flow rate 12 L·min^−1^; drying gas temperature 350°C; Nebulizer 35 psi; mass analyzer range was from *m*/*z* 50 to 1250 amu. All the data were processed using Data Analysis for 6300 series Ion Trap LC/MS (Version 3.4).

### 2.4. Different Types of Fingerprints

In order to investigate whether different types of fingerprints characterized by different modes have notable differences in characterization of XKS tablet samples, four types of fingerprints were applied, including single wavelength fingerprint, 3D fingerprint, maximum absorption wavelength fingerprint, and multiwavelength fingerprint. The four types of fingerprints were developed as described in Additional File 1 in Supplementary Material available online at http://dx.doi.org/10.1155/2015/589654.

### 2.5. Data Analysis

HPLC-DAD data of the 71 batches of samples were obtained from Agilent ChemStation software. The software “Similarity Evaluation System for Chromatographic Fingerprint of Traditional Chinese Medicine (Version 2004A)” recommended by CFDA (The China Food and Drug Administration) was used to align the fingerprints of different batches and to simultaneously calculate similarity between samples. MATLAB 2009B (Math Works, Inc., Natick, MA, USA) was used for handling chromatographic data matrices, calculating the Mahalanobis distance and carrying out hierarchical clustering analysis (HCA) principle component analysis (PCA) and statistical test (i.e., *t*-test and *F* test).

### 2.6. Similarity Analysis

About similarity calculation, one is cosine of vector angle (*r*_cos_) that is often used; the other is correlation coefficient (*r*). Both *r*_cos_ and *r* were calculated for similarity evaluation of chromatographic fingerprints among different batches of samples.

### 2.7. Hierarchical Component Analysis (HCA)

The goal of HCA is to find an optimal grouping between samples. The HCA result is usually displayed in a dendrogram for interpretation [14]. Ward's method was used for HCA due to relatively powerful performance compared with other methods [15].

### 2.8. Mahalanobis Distances Analysis

Mahalanobis distance is a distance measure in statistics and takes into account the correlated variables in data set [16], which can be written as

(1) $$\begin{array}{c} D_{i}^{2}=\left(\;x_{i}-\overset{-}{x}\;\right)S^{-1}{\left(\;x_{i}-\overset{-}{x}\;\right)}^{\prime }, \end{array}$$

where **x**_*i*_, $\overset{-}{x}$, and **S** ^−1^ are the *i*th sample, the mean vector (i.e., the centroid of class), and the variance-covariance matrix of a class, respectively. According to Mahalanobis distance, the unknown samples can be classified as acceptable or unacceptable for the training set at a certain probability level [17, 18]. Critical values at a certain probability level (*α*) are obtained by *F* distribution [17]. The Mahalanobis distance of a sample was compared with critical value to determine whether the difference of the sample relative to the mean of the training set was significant or not. The 95% confidence level was selected in this study. The sample having a probability level in the range from 1.0 to 0.05 is classified as a member of training set. The sample having a probability level in the range from 0.05 to 0 is classified as an outlier.

## 3. Results

### 3.1. Validation of HPLC Fingerprint Method

Since the single wavelength fingerprint was often used in practice, the validation of HPLC fingerprint method for XKS tablet was performed on the basis of this type of fingerprint. The same sample solution was run six times to obtain HPLC method precision. The relative standard deviation (RSD) values of common peak areas and relative retention time of common peaks were in the range of 0.6–3.5% and 0.3–1.2%, respectively. The repeatability was estimated by running five independently prepared XKS tablet samples, which were from the same batch, and the corresponding RSD values of common peak areas and relative retention time of common peaks were lower than 1.8% and 1.4%, respectively. A sample solution placed over 3 days in room temperature was employed to carry out the stability test. The corresponding RSD values of common peak areas and relative retention time of common peaks were less than 3.5% and 2.3%, respectively. The results of validation suggested that the developed HPLC fingerprint method was satisfactory and applicable to sample analysis.

### 3.2. Similarity Analysis of Different Types of Fingerprints

**Figure 1:**
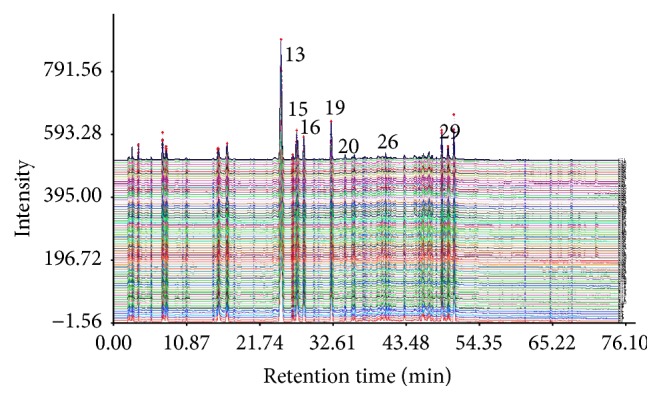
Typical fingerprints (single wavelength fingerprints) of 71 batches of XKS tablet samples after peak alignment.

The typical fingerprints (single wavelength fingerprints) of 71 batches of XKS tablet samples after peak alignment were displayed in Figure 1.

Similarity analysis was employed to compare the four types of fingerprints. The similarity between the fingerprint of each sample and the standard fingerprint (i.e., the mean fingerprint of 71 batches of samples) was shown in Additional File 2 (Supplementary Figures 1 and 2). There are no apparent differences found in similarity trend along with the sample distribution for different types of fingerprints. Although fingerprints are based on different characterization modes, the fingerprints of different batches of samples have a high similarity to the standard fingerprint (all above 0.94 based on *r* or *r*_cos_). According to the literature [19], the value higher than 0.95 means that a good similarity is achieved. In addition to the maximum wavelength fingerprint, the other three types of fingerprints are considered to have good similarity for different batches of samples. The similarities calculated by *r* and *r*_cos_ were very close for each sample. The range and the degree of dispersion of similarities based on four types of fingerprints were summarized in Table 1.

According to Table 1, the maximum wavelength fingerprint has the largest range and the dispersion degree of similarity as compared with the other three types of fingerprints. Because this type of fingerprint is characterized with the data at maximum absorbance wavelength, the sensitivity is relatively high. The 3D fingerprint is very close to the single wavelength fingerprinting in the range and the dispersion degree of similarity. Among four types of fingerprints, multiwavelength fingerprint exhibits the minimum range and dispersion degree of similarity due to the quite close distribution of multiactive ingredients in different batches of samples.

Up to now, the single wavelength fingerprint is the most used in practice. In this type of fingerprint, the particular wavelength was generally selected to detect as many chromatographic peaks as possible. The 3D fingerprint for characterizing each sample contained rich information on components; however, redundant information was also present. Additionally, poor resolution and unstable baseline appeared in some wavelengths, making the subsequent data processing (such as integration and peak alignment) time-consuming, difficult, and complicated. The multiple-wavelength fingerprint could have good reflection on characteristic components but lost some useful information on other constituents. The maximum wavelength fingerprint was a satisfactory type of fingerprint and could better reflect the differences in chemical composition between samples. However, it was not easy to get the maximum absorbance fingerprint, because some complicated and time-consuming data handling methods or special ChemStations (such as Waters Empower ChemStation) should be necessary. According to the comparison mentioned above, the single wavelength fingerprint seems to be still the most practical, popular, and easy-handling fingerprint pattern for quality evaluation of herbal medicine product. Considering fingerprint applicability and representativeness, the single wavelength fingerprint was selected for further investigation.

### 3.3. Hierarchical Clustering Analysis (HCA)

**Figure 2:**
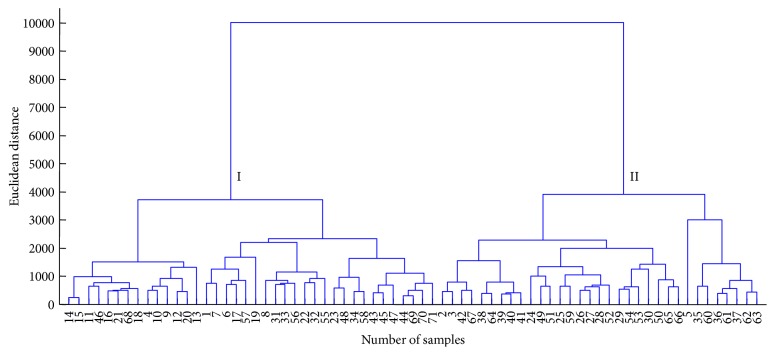
Dendrogram of hierarchical cluster analysis of different batches of XKS tablet samples using Ward's method.

HCA was used to find relatively analogous clusters of 71 batches of samples.  Figure 2 shows the dendrogram of HCA.

Overall, the dendrogram revealed two clear clusters, suggesting that there were apparent class differences in different batches of XKS tablet samples. Although high similarity between samples was acquired (Supplementary Figure 2), the class differences seemed to be significant.

### 3.4. Mahalanobis Distances Analysis

In order to determine whether or not the differences between batches of XKS tablet samples were statistically significant, Mahalanobis distance method was employed. For calculation of Mahalanobis distance, the reference class should be constructed first. Good quality samples should be candidates of the reference class. In our study, a sample having larger peak areas in its fingerprint was selected as the member of the reference class due to large peak area generally indicating high content of component.

Sample 5 was first selected as a reference sample for development of the reference class, because most of peak areas in sample 5 fingerprint are larger as compared to other batches of samples. The Euclidian distances between other batches of samples and sample 5 were then calculated for evaluation of sample closeness. The reference class was developed by choosing the closest 10 batches of samples plus sample 5; therefore, 11 samples were involved in the reference class according to the requirement of the documents on the development of fingerprint. The mean fingerprint of the samples in the reference class was also calculated as the reference fingerprint. With respect to the centroid of the reference class, the Mahalanobis distance and its probability level of each sample in the reference class are summarized in Table 2.

The results reveal that all sample fingerprints in the reference class have nonsignificant differences as compared to the reference fingerprint.

**Figure 3:**
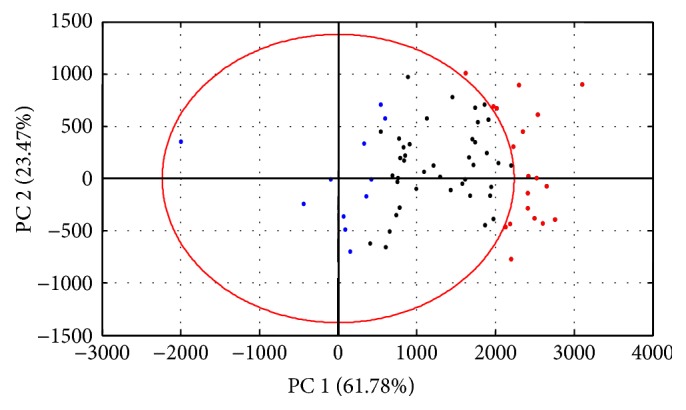
PCA projection plot for other batches of samples relative to the samples in the reference class. The blue dots represent the samples in the reference class; the black dots refer to the batches of samples with no significant differences to the centroid of the reference class; the red dots denote the batches of samples having significant differences to the centroid of the reference class. Red ellipse is drawn at 95% probability (*α* = 0.05).

The Mahalanobis distances between the fingerprints of other batches of samples and the reference fingerprint were also calculated and shown in Figure 3. The differences in fingerprints were identified by *F* test for significance [17].

There are 19 batches of samples having significant differences with respect to the centroid of the reference class determined by Mahalanobis distances, indicating that their fingerprints may have large differences in peak areas relative to the reference fingerprint.

**Figure 4:**
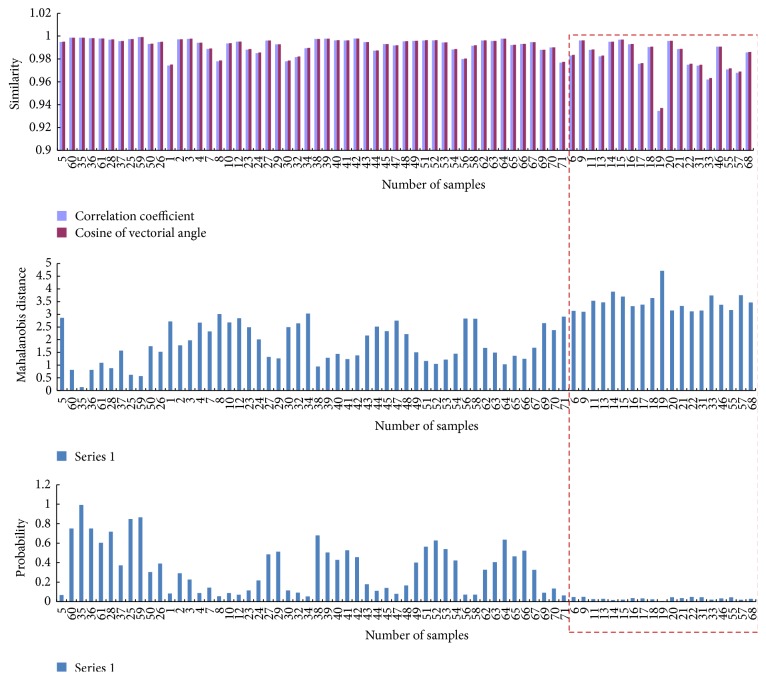
Similarities, Mahalanobis distances, and corresponding probabilities between the fingerprints of different batches of samples and the reference fingerprint. The fingerprints having significant differences to the reference fingerprint are shown in the red dotted box region.

To explore the relationship between similarity (both cosine of vector angle and correlation coefficient) and Mahalanobis distance, similarity, Mahalanobis distance, and corresponding probability of each batch fingerprint relative to the reference fingerprint are shown in Figure 4.

Generally, the higher the similarity is, the smaller the Mahalanobis distance is. The relationship between similarity and Mahalanobis distance seems ambiguous (see Figure 4). However, although the fingerprints of some samples (such as samples 9, 15, and 20, and their *r* and *r*_cos_ are both above 0.99) have high similarity to the reference fingerprint, their corresponding Mahalanobis distances are still larger, which result in significant differences (*α* < 0.05). Nonconsistent results were found in evaluation of closeness by similarity and Mahalanobis distance. Notice that higher similarity does not exactly indicate small Mahalanobis distance, while lower similarity indicates larger difference in Mahalanobis distance.

According to (1), the Mahalanobis distance is actually based on the differences in peak areas between fingerprints. For *r* or *r*_cos_, although the chromatographic peak areas are used in similarity calculation, similarity indicates a measure of the closeness of the overall shapes of the compared fingerprint profiles. When the shapes of the two HPLC fingerprints are very close, high similarity can be obtained even if the peak areas in the compared fingerprints are quite different (e.g., the peak areas vary greatly with different injection volumes in different fingerprints, but the similarity is still high owing to close shapes of fingerprint profiles). If the difference in peak area is very great and can significantly affect the shape of the compared fingerprints, the similarity between the fingerprints will change accordingly.

Therefore, the Mahalanobis distance takes the difference of peak area into account, while similarity emphasizes the overall shape of the fingerprint profile. In some cases, although the fingerprints of some batches showed high similarity to the reference fingerprint, their differences in peak areas were still significant based on Mahalanobis distances. Therefore, the Mahalanobis distance could reflect the inconsistency between batches of samples relatively better than similarity, and the corresponding probability level was also estimated for assessing whether the inconsistency between fingerprints was significant or not.

### 3.5. Recognition and Identification of the Significantly Different Peaks

Chromatographic peaks in a HPLC fingerprint are used to characterize components in a sample. The peak area is associated with the content of component. If peak area has large difference in various batches of samples, quality consistency will be strongly affected. Therefore, it is quite necessary to find the significant different peaks across the fingerprints of batches. The Mahalanobis distance was used to detect whether there are significant differences between the sample fingerprints and the reference fingerprint. However, it is not easy to find the significantly different peaks in a sample fingerprint by Mahalanobis distance. In our study, the *t*-test was employed to detect the peaks that had significant differences. Because common peaks are common features of different batches, differences in areas of common peaks will have great impact on quality consistency. Moreover, for each sample fingerprint, the common peak areas also accounted for above 81% of the total peak areas. Therefore, only common peaks were identified by *t*-test for significantly different batches determined by Mahalanobis distance. More attention should be paid to the significantly different common peaks for the quality control of XKS tablet.

The reference class could be regarded as the training set. To carry out the *t*-test on the peaks in a sample fingerprint, the variable *z*_*i*_ was calculated according to

(2) $$\begin{array}{c} z_{i}=\frac{\left(x_{i}-{\overset{-}{x}}_{i}\right)}{s_{i}}, \end{array}$$

where *x*_*i*_ referred to the *i*th chromatographic peak area in a fingerprint of sample, and ${\overset{-}{x}}_{i}$ and *s*_*i*_ were the average peak areas and the corresponding standard deviations of peak *i* in the fingerprints of the samples in the training set (i.e., the reference class), respectively. The *t*_*i*_ was calculated by [17]

(3) $$\begin{array}{c} t_{i}=z_{i}{\left[\;\frac{n}{\left(n+1\right)}\;\right]}^{1/2}, \end{array}$$

where *n* was the number of samples in the training set (*n* = 11 in the present study).

The *t*_*i*_ was compared with the *t*_crit_ given by Student's *t* distribution to determine whether the difference between *x*_*i*_ and ${\overset{-}{x}}_{i}$ was significant or not. Although, *t*-test was calculated with chromatographic peak area, the result indirectly denoted the differences in component contents of different batches of samples, because, in the linear range, a chromatographic peak area was expressed by

(4) $$\begin{array}{c} x_{i}=f_{i}c_{i}+b_{i}, \end{array}$$

where *x*_*i*_ and *c*_*i*_ referred to the chromatographic peak area of peak *i* and its corresponding concentration (or content), respectively. *f*_*i*_ and *b*_*i*_ denoted the response factor and the intercept of the calibration curve, individually. Then, (2) combined with (4) could be written as

(5) zixi−x−isi=fici−c−ifi1/n−1∑i=1nci−c−i21/2=ci−c−isci,

where ${\overset{-}{c}}_{i}$ and *s*_*c*___*i*__ represented the average content and the standard deviation of *i*th component concentrations in the samples of the reference class. So, according to (5), the value of *z*_*i*_ based on peak area was identical to that based on component content for peak *i*.

**Figure 5:**
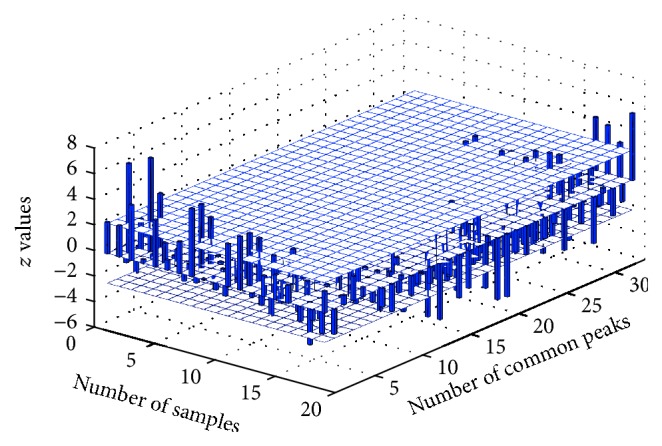
*z* values of common peaks in 19 batches of samples having significant differences based on Mahalanobis distance analysis. The blue bars represent the *z* values of 33 common peaks. *z*_crit_ plane is based on the *t*_crit_ value given by *t*-test at 95% confidence level (*α* = 0.05).

Figure 5 shows the *z* values of common peaks in 19 significantly different batches of samples determined by Mahalanobis distance analysis.

Among the 19 batches of samples, most of common peak areas with significant differences identified by *t*-test are the small ones in peak areas, which indicate that the amounts of these components may be lower than the average contents of the corresponding components in the samples of the reference class.

**Figure 6:**
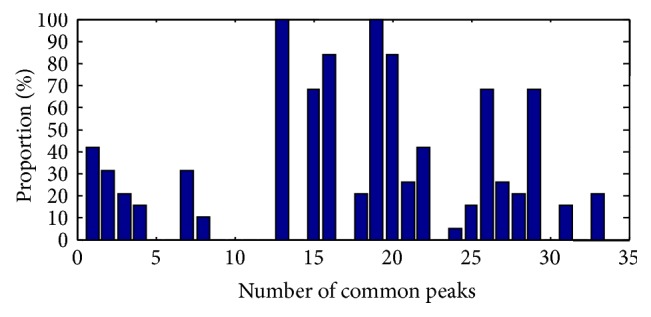
The frequency proportion of the number of significantly different common peaks to 19 batches of samples.

Figure 6 shows the frequency proportion of significantly different common peaks based on *t*-test versus 19 batches of samples. The results clearly reveal the common peaks 13 and 19 having the highest frequency percentage that occurred in the 19 batches of samples.

**Figure 7:**
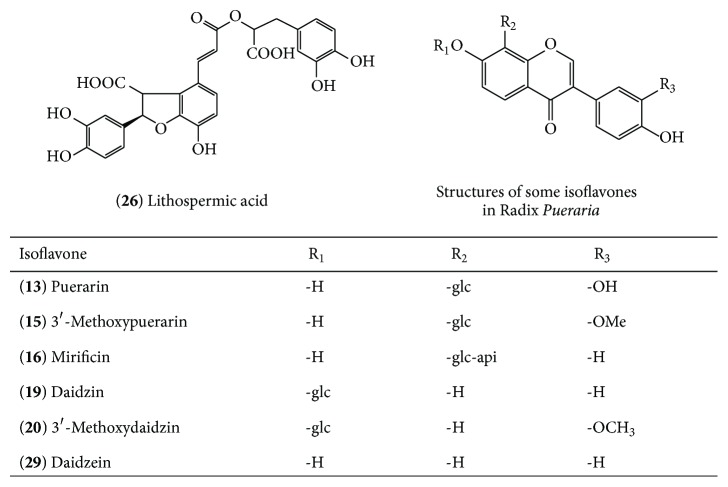
Chemical structures corresponding to seven common peaks.

The components corresponding to the common peaks with high frequency percentage may have large differences in amount. These common peaks would strongly affect the consistency of batch quality. HPLC/IT-MS^*n*^ was employed to identify seven of them (peaks** 13**,** 15**,** 16**,** 19**,** 20**,** 26,** and** 29**) which displayed high frequency proportion in Figure 6 and are marked in Figure 1. In the ESI-MS^*n*^ experiment, the molecular weight of each peak and some fragments could be obtained. In negative ion ESI mode, the peaks were detected according to the deprotonated molecules ([M-H]^−^ and/or [M-H + HCOOH]^−^). Except peak** 26**, which was identified as lithospermic acid (characterized based on the retention time, UV spectrum, and its MS data compared with literature [20]), the other six interesting peaks were tentatively identified as isoflavonoids. The diagnostic ions of isoflavonoids are the loss of 162 Da and 120 Da in molecular weight, which are related to the O- and C-glycoside isoflavones [21, 22], respectively. The possible structures of peaks** 13**,** 15**,** 16**,** 19**,** 20**,** 26,** and** 29** were characterized based on their retention times, UV spectra, and their MS data compared with literatures. Using peak** 15** as an example, the fragmentation of [M-H]^−^ ion at *m*/*z* of 445 gives product ions at *m*/*z* 325 [M-H-120]^−^, 297 [M-H-120-CO]^−^, and 282 [M-H-120-CO-CH_3_]^−^, which indicate that peak 15 has a -OCH_3_ group in the aglycone skeleton and the neutral loss of 120 Da characteristic of C-glycosides. Compared with the literature [23], peak** 15** was identified as 3′-methoxypuerarin. More information about these seven peaks and their structures are shown in Table 3 and Figure 7, respectively.

According to MS results, lithospermic acid and six isoflavonoids as characteristic markers may have strong contribution to the inconsistency of batches of XKS tablet samples. The isoflavonoids were the main active ingredients of* Pueraria lobatae* Radix, suggesting that the* Pueraria lobatae* Radix could have large fluctuations in the production of XKS tablet. Consequently, in order to improve the consistency of different batches of XKS tablet, more attention should be paid to the quality of* Pueraria lobatae* Radix, including selection of good quality of raw materials, improvement of manufacturing technologies, and enhancement of process control level.

**Figure 8:**
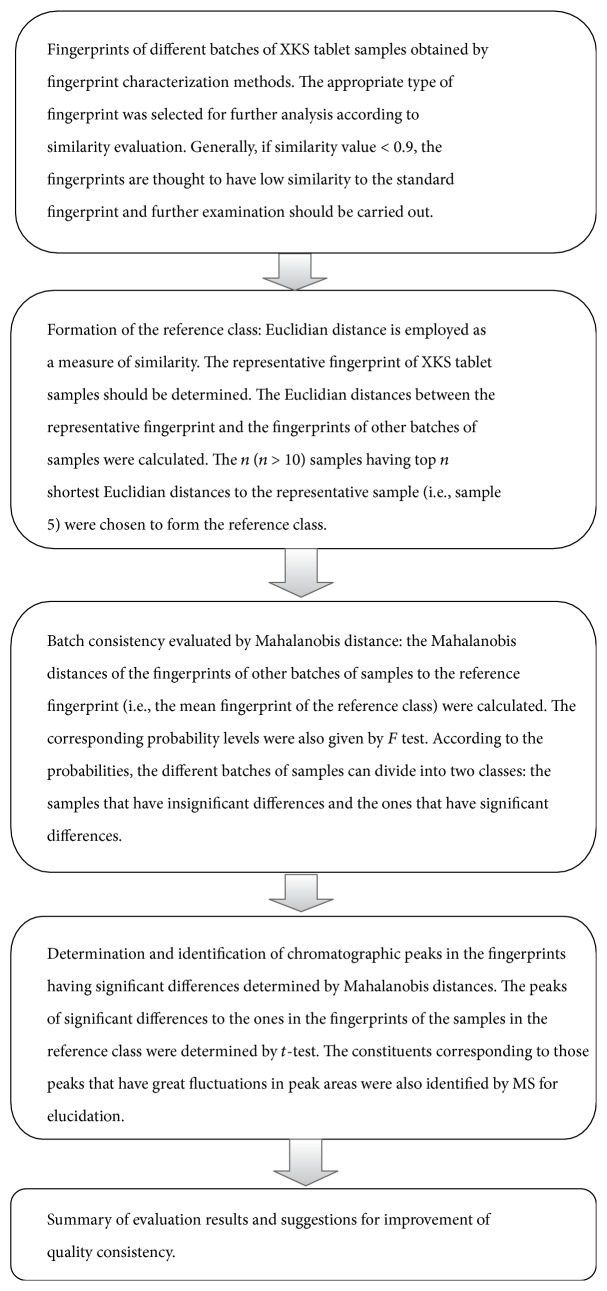
A strategy for the consistency evaluation of different batches of XKS tablet samples.

To find a rational way to evaluate the consistency of different batches of XKS tablet samples, a strategy was tentatively proposed (see Figure 8).

## 4. Conclusion

The useful strategy was proposed for the solution of the routine issue existing in the consistency evaluation of chromatographic fingerprints of XKS tablet samples. Although high similarity occurred in some batches of samples, the significant differences between samples could still be found by Mahalanobis distance. The chromatographic peaks with significant differences in peak areas determined by *t*-test could suggest that the corresponding constituents would have significant differences in content, which resulted in affecting the consistency of quality. Our study demonstrated that the proposed strategy based on the combination of HPLC fingerprints and chemometric methods would provide a useful way to assess the quality consistency of XKS tablet. This strategy would be a practical reference for the preparations of other TCMs.

## Supplementary Material

Online Supporting Information 1: Development of the four typical fingerprints included single wavelength fingerprint, 3D fingerprint, Maximum absorption wavelength fingerprint, and multi-wavelength fingerprint.
Supplemental Figure 1. Similarities between the fingerprint of each sample and the standard fingerprint.
Supplemental Figure 2. Fingerprint of XKS and the five individual herbals. Fingerprint of XKS was the mean fingerprint derived from the fingerprints of 71-batch samples by average method generated using software named “the similarity evaluation system for TCM”. The common peaks in XKS fingerprint were assigned to each crude herb. For the 35 common peaks, 13 of them were from *Pueraria lobatae* radix (Chinese name Gegen, GG), 7 of them were from *Salviae Miltiorrhizae* Radix et Rhizoma (Chinese name Danshen, DS), 13 of them were form *Crataegi* Fructus (Chinese name Shanzha, SZ), and 2 of them were from *Aucklandiae* Radix (Chinese name Muxiang, MX). We cannot found any peaks belong to *Radix Notoginseng* in the fingerprint of XKS tablet, since for *Notoginseng* Radix et Rhizoma (Chinese name Sanqi, SQ), the major bioactive constituents are saponins. For this type of constituents, the maximum UV absorbance wavelength is 203 nm, thus, we built another quantification method for the quality control of the saponins in XKS tablet (data not shown). All the common peaks were identified by LC-MS/MS. The peaks were assigned to each crude herb based on both the MS information and the retention times (data not shown).

## Figures and Tables

**Table 1:** Range and dispersion degree of similarities based on four types of fingerprints.

	*r*_max_	*r*_min_	*r*_max_ − *r*_min_	RSD_*r* (%)	*r*_cos_max_	*r*_cos_min_	*r*_cos_max_ − *r*_cos_min_	RSD_*r*_cos_ (%)
3D fingerprint	0.9977	0.9581	0.0396	0.6170	0.9977	0.9593	0.0384	0.5995
Multiple wavelength fingerprint	0.9985	0.9790	0.0195	0.3052	0.9986	0.9794	0.0192	0.2993
Maximum wavelength fingerprint	0.9980	0.9463	0.0517	0.8266	0.9981	0.9492	0.0489	0.7832
Single wavelength fingerprint	0.9982	0.9567	0.0415	0.6425	0.9983	0.9587	0.0396	0.6130

**Table 2:** Mahalanobis distance and its probability level of each sample in the reference class.

Number of sample	Mahalanobis distance	Probability level
5	2.862	0.068
60	0.811	0.751
35	0.131	0.992
36	0.813	0.750
61	1.091	0.603
28	0.876	0.717
37	1.570	0.371
25	0.614	0.847
59	0.571	0.865
50	1.741	0.304
26	1.523	0.391

**Table 3:** Identification of common peaks by HPLC/IT-MS^*n*^.

Number	Retention time (min)	Assigned identity	UV *λ*_max_ (nm)	(−)ESI-MS^*n*^	HPLC-ESI-MS^*n*^ (*m*/*z*) (% base peak)
13	24.9	Puerarin	250, 310	415 [M-H]^−^	MS^2^ [415]: 295 (100), 267 (82) MS^3^ [415→295]: 267 (100)

15	27.5	3′-Methoxypuerarin	250, 300	445 [M-H]^−^	MS^2^ [445]: 325 (100), 297 (21), 282 (21), MS^3^ [445→325]: 297 (100), 282 (88)

16	28.5	Mirificin	250, 310	547 [M-H]^−^	MS^2^ [547]: 295 (100), 267 (82) MS^3^ [547→295]: 267 (100)

19	32.4	Daidzin	250, 310	461 [M-H+HCOO^−^]^2−^	MS^2^ [461]: 415 (37), 253 (100) MS^3^ -

20	34.5	3′-Methoxydaidzin	250, 310	491 [M-H+HCOO^−^]^2−^	MS^2^ [491]: 445 (21), 283 (100), 268 (4.7) MS^3^ [491→283]: 268 (100), 240 (2)

26	40.9	Lithospermic acid	254, 310	537 [M-H]^−^	MS^2^ [537]: 493 (28), 295 (100) MS^3^ [537→295]: 277 (100)

29	49.9	Daidzein	250, 310	253 [M-H]^−^	MS^2^ [253]: 225 (100), 211 (22), 135 (45), 91 (22) MS^3^ -

(−) in negative scan mode in HPLC/IT-MS^*n*^.
