# Supplementary material for: A Useful Strategy to Evaluate the Quality Consistency of Traditional Chinese Medicines Based on Liquid Chromatography and Chemometrics
Source: J Anal Methods Chem. 2015 Nov 4;2015:589654. doi: 10.1155/2015/589654 (PMC4649102; doi:10.1155/2015/589654)
Supplement: Supplementary file 1 — Online Supporting Information 1: Development of the four typical fingerprints included single wavelength fingerprint, 3D fingerprint, Maximum absorption wavelength fingerprint, and multi-wavelength fingerprint. Supplemental Figure 1. Similarities between the fingerprint of each sample and the standard fingerprint. Supplemental Figure 2. Fingerprint of XKS and the five individual herbals. Fingerprint of XKS was the mean fingerprint derived from the fingerprints of 71-batch samples by average method generated using software named “the similarity evaluation system for TCM”. The common peaks in XKS fingerprint were assigned to each crude herb. For the 35 common peaks, 13 of them were from Pueraria lobatae radix (Chinese name Gegen, GG), 7 of them were from Salviae Miltiorrhizae Radix et Rhizoma (Chinese name Danshen, DS), 13 of them were form Crataegi Fructus (Chinese name Shanzha, SZ), and 2 of them were from Aucklandiae Radix (Chinese name Muxiang, MX). We cannot found any peaks belong to Radix Notoginseng in the fingerprint of XKS tablet, since for Notoginseng Radix et Rhizoma (Chinese name Sanqi, SQ), the major bioactive constituents are saponins. For this type of constituents, the maximum UV absorbance wavelength is 203 nm, thus, we built another quantification method for the quality control of the saponins in XKS tablet (data not shown). All the common peaks were identified by LC-MS/MS. The peaks were assigned to each crude herb based on both the MS information and the retention times (data not shown). [file 589654.f1.doc]

**Online Supporting Information**

**Development of the four typical fingerprints**

### single wavelength fingerprint

The single wavelength fingerprint is the most commonly used in routine applications. In our present work, the single wavelength fingerprint was detected at a wavelength of 278 nm, because more chromatographic peaks were detected and resolution was also satisfactory as compared with other wavelengths. The fingerprints constructed based on the four methods were compared by similarity analysis to evaluate which type of fingerprint was superior in the reflection of the differences between different batches of XKS samples. As the detailed fingerprint methods were not the focus of this work, only brief description of them was exhibited as follows.

### 3D fingerprint

3D fingerprint, also known as the holographic fingerprint, is constructed by information data based on diode array detector (DAD). In this study, we made some modification to the original 3D fingerprint method. The multivariate absorption data from 16 wavelengths (from 210 nm to 360 nm at intervals of 10 nm) was used to replace the absorption data from 106 wavelengths (from 190 nm to 400 nm at intervals of 2 nm), because the ultraviolet (UV) absorption below 210 nm was vulnerable to interference, while for XKS samples the UV absorption above 360nm was very low, thus the scope from 210 nm to 360 nm was selected. Additionally, after carefully investigation, the intervals between wavelengths expanded to 10nm could significantly reduce the calculation time. In order to avoid the loss of some useful information, the UV absorption at 278 nm and 286 nm were also included in 3D fingerprint. Then, the peaks in each fingerprint detected at each wavelength was integrated and aligned. All data process and calculation were based on peak area in fingerprint. For each sample, a data matrix of dimensions 18×175 (number of wavelengths×number of peak areas) was acquired.

### Maximum absorption wavelength fingerprint

In the maximum absorption wavelength fingerprint, the absorption data of each chemical component at its maximum UV absorption wavelength was recorded on a single chromatogram (i.e. fingerprint). However, to get the maximum absorption wavelength fingerprint is not an easy task, because some complex data handling methods or special chemstations are needed. In our work, the maximum absorption wavelength of each peak was extracted using the codes ofMatlab developed by ourselves. Then, the area of each peak was integrated at its maximum absorption wavelength and aligned. Because the maximum wavelength fingerprint maximized the ultraviolet absorption sensitivity of each peak, it could maximize the response to the complex component composition information of TCMs.

### Multi-wavelength fingerprint

In multi-wavelength fingerprint, the wavelengths were selected according to the UV characteristic absorption peaks of the major active components. In the prescription of XKS, the five herbs play different roles according to theory and hypothesis of TCMs. Among them, *Salviae Miltiorrhizae* Radix et Rhizoma and *Pueraria lobatae* radix are major herbs in content, and they play the important role of “King” and “minister”, respectively. Both salvianolic acids in *Salviae Miltiorrhizae* Radix et Rhizoma and isoflavonoids in *Pueraria lobatae* radix were well studied for their significant activities in treatment of coronary heart and cerebrovascular disease .Thus, four UV characteristic absorption wavelengths (280 nm, 286 nm and 330 nm for salvianolic acids; 250 nm for the isoflavonoids) were selected for the construction of the multi-wavelength fingerprint. Therefore, four fingerprints were detected to characterize one sample. The peaks in each fingerprint at each wavelength were integratedand aligned. For each sample, a data matrix of dimensions 4×175 (number of wavelengths×number of peak areas) was acquired.

### References

1. X. Wang, S.L. Morris-Natschke, and K.H. Lee., “New developments in the chemistry and biology of the bioactive constituents of tanshen,” Medicinal Research Reviews, vol 27, no. 1, pp. 133-148.

2. Y.X. Chang, X.P. Ding, J. Qi, et al., “The antioxidant-activity-integrated fingerprint: An advantageous tool for the evaluation of quality of herbal medicines,” Journal of Chromatography A, vol. 1208, no. 1-2, pp. 76-82.

3. R.L.Cai, M. Li, S.H. Xie, et al., “Antihypertensive effect of total flavone extracts from Puerariae Radix,” Journal of Ethnopharmacology, vol. 133, no. 1, pp.177-183.

4. K.H. Wong, G.Q. Li, K.M. Li, “Razmovski-Naumovski, V., Chan, K.; Kudzu root: Traditional uses and potential medicinal benefits in diabetes and cardiovascular diseases,” Journal of Ethnopharmacology, vol. 134, no. 3, pp. 584-607.

**Online Supporting Information**


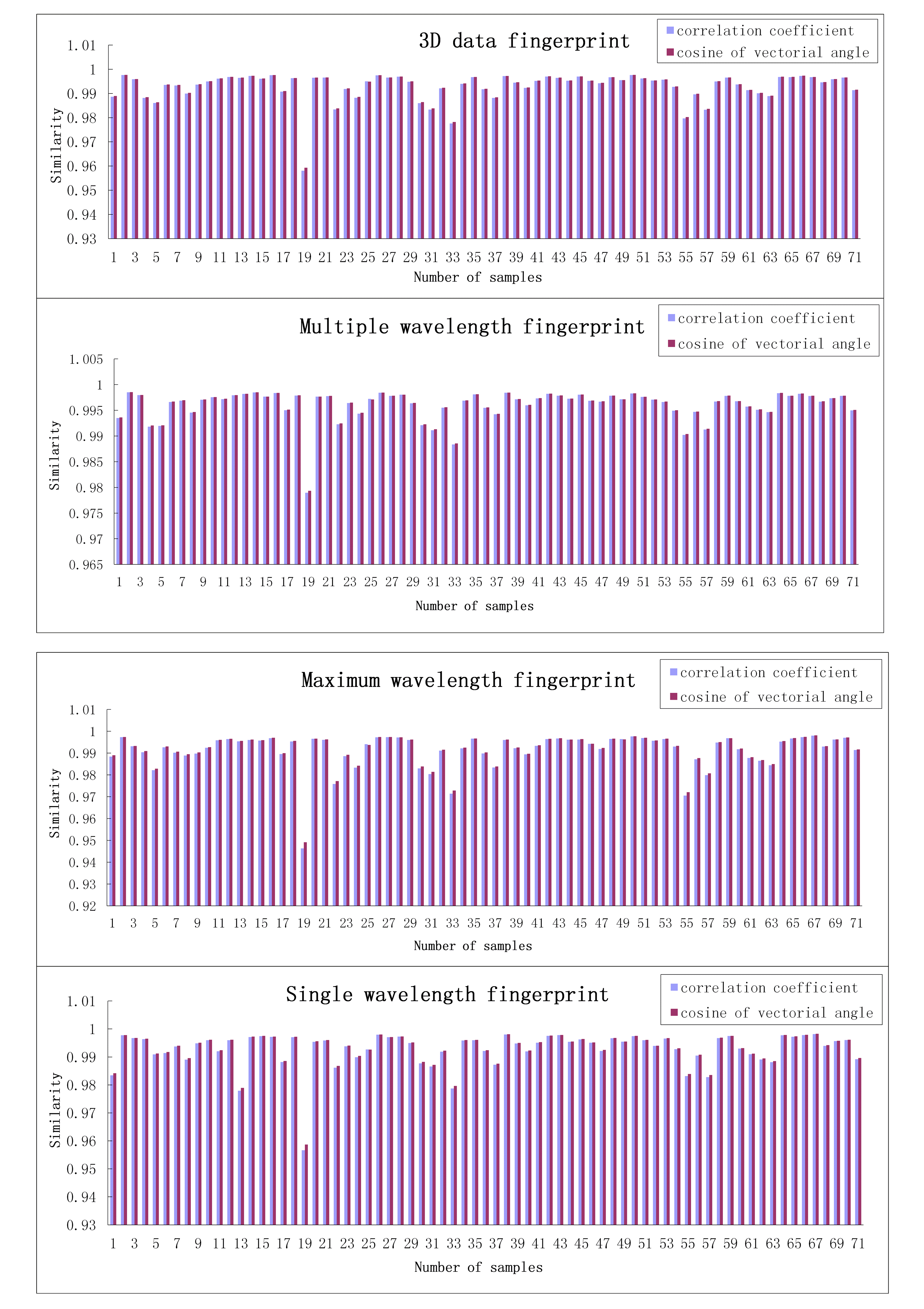


**SupplementalFigure 1.** Similarities between the fingerprint of each sample and the standard fingerprint.


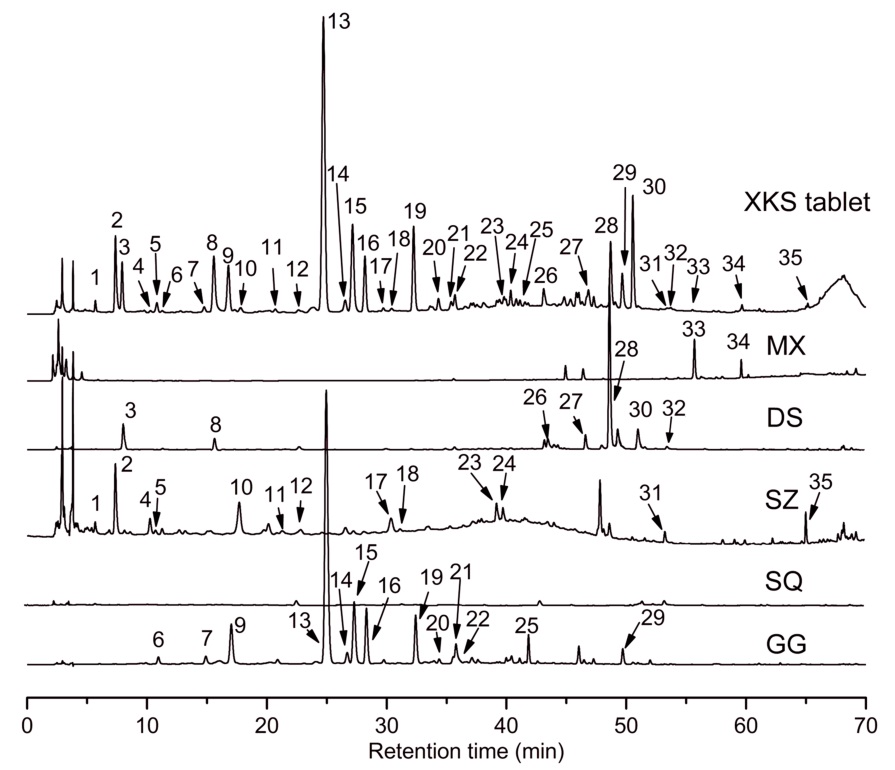


**Supplemental Figure 2.** Fingerprint of XKS and the five individual herbals. Fingerprint of XKS was the mean fingerprint derived from the fingerprints of 71-batch samples by average method generated using software named “the similarity evaluation system for TCM”. The common peaks in XKS fingerprint were assigned to each crude herb. For the 35 common peaks, 13 of them were from *Pueraria lobatae* radix (Chinese name Gegen, GG), 7 of them were from *Salviae Miltiorrhizae* Radix et Rhizoma (Chinese name Danshen, DS), 13 of them were form*Crataegi*Fructus (Chinese name Shanzha, SZ), and 2 of them were from *Aucklandiae* Radix (Chinese name Muxiang, MX). We cannot found any peaks belong to *Radix Notoginseng* in the fingerprint of XKS tablet, since for *Notoginseng* Radix et Rhizoma (Chinese name Sanqi, SQ), the major bioactive constituents are saponins. For this type of constituents, the maximum UV absorbance wavelength is 203 nm, thus, we built another quantification method for the quality control of the saponins in XKS tablet (data not shown). All the common peaks were identified by LC-MS/MS. The peaks were assigned to each crude herb based on both the MS information and the retention times (data not shown).
